# Morphometrics for sports mechanics: Showcasing tennis racket shape diversity

**DOI:** 10.1371/journal.pone.0263120

**Published:** 2022-01-31

**Authors:** Robyn A. Grant, Luca Taraborrelli, Tom Allen

**Affiliations:** 1 Department of Natural Sciences, Manchester Metropolitan University, Manchester, United Kingdom; 2 Department of Engineering, Manchester Metropolitan University, Manchester, United Kingdom; University of Bologna, ITALY

## Abstract

Tennis racket design has changed from its conception in 1874. While we know that modern tennis rackets are lighter and have larger heads than their wooden predecessors, it is unknown how their gross shape has changed specifically. It is also unknown how racket shape is related to factors that influence performance, like the Transverse and Polar moments of inertia. The aim of this study was to quantify how tennis racket shape has changed over time, with a view to furthering our understanding of how such developments have influenced the game. Two-dimensional morphometric analysis was applied to silhouettes extracted from photographs of 514 rackets dating from 1874 to 2017. A principal component analysis was conducted on silhouette outlines, to allow racket shape to be summarised. The rackets were grouped by age and material for further analysis. Principal Component 1 accounted for 87% of the variation in racket shape. A pairwise Pearson’s correlation test indicated that head width and length were both strongly correlated to Principal Component 1 (r = 0.916 & r = 0.801, p-values<0.001). Principal Component 1 was also correlated to the Polar (r = 0.862, p<0.001) and Transverse (r = -0.506, p<0.001) moments of inertia. Racket age and material had a medium (p<0.001, η2p = 0.074) and small (p = 0.015, η2p = 0.017) effect on Principal Component 1, respectively. Mean racket shapes were also generated from the morphometric analyses for the material and age groupings, and we consider how these shape changes may have influenced performance and injury risk. These mean shape groupings could support the development of models, such as finite element analysis, for predicting how historical developments in tennis equipment have affected performance and injury risk.

## 1. Introduction

The shape of an object can influence its mechanical properties, including its stiffness, centre of mass and moments of inertia (MOI) [1]. In many areas of engineering and biomechanics, simple geometries, like beams, plates, and cylinders, are used to approximate shape [2,3]. Simplifying object shape commonly occurs in studies of sports equipment mechanics [3], where implements like bats [4,5] and rackets [6–8] are often modelled as beams. For example, Haake et al. [6] modelled lawn tennis rackets as beams and predicted that developments in equipment, since the origins of the game in the 1870s, have allowed players to serve almost 20% faster. However, evidence suggests that shape needs to be better incorporated into mechanical models of objects [4,5].

Indeed, the shape of lawn tennis rackets has changed considerably over time. Early tennis rackets were asymmetric and wooden with small heads, but they are now made from fibre-polymer composites and are symmetric with relatively large heads [6,9–12]. Taraborrelli et al. [10] collected a large dataset of measurements on over 500 tennis rackets, dating from 1874 to 2017. They summarised their measurements using a principal component analysis (PCA) [13], which is an established method for reducing the dimensionality of a large dataset. Principal component analysis combines the original variables in the dataset into an equal number of principal components. The principal components are numbered in ascending order, with the first one (Principal Component 1 (PC1)) capturing the most information in the dataset, and the last one capturing the least. By keeping the principal components that capture the most information in the dataset, and discarding the others, the dimensionality of a dataset can be reduced without losing much information.

Within the first three principal components (of 12), Taraborrelli et al. [10] captured 64% of the information from 12 measurements on each racket in the dataset, including frame dimensions, vibration characteristics and inertial properties. The first principal component accounted for about a third of the variance in the racket measurements, and was largely affected by racket material. They also found head width and head length to correlate best with PC1, which indicates an interaction between racket shape and material over time. It would now be interesting to see if more variation of the racket can be captured using geometric shape analysis, rather than these discrete racket measurements.

Mechanical models are beneficial for investigating the effect of racket design, particularly shape and material, on performance and injury risk, as they allow control over parameters that are inherently variable and hard to measure during physical experiments. Beam models are, however, unable to account for the observed complexities in racket shape, such as different head widths or asymmetry that are known to vary across rackets [10] and could affect performance and injury risk. It has been suggested that the lower mass [12,14] and larger head size [12] of modern tennis rackets increases the risk of upper extremity injuries, including overuse injuries to the elbow [15]. Beam models are particularly unable to capture impacts away from the longitudinal axis that force extension of the wrist [16].

Finite element models can faithfully capture the shape of an object [2,17–23], with the potential to systematically investigate possible associations between tennis racket design, performance, and injury risk. However, developing geometrically faithful finite element models of tennis rackets is time-consuming, so it is inefficient and impractical to apply this technique to many samples. Developing a way to summarise key shape changes, or “mean” racket shapes, would be a useful first step in reducing individual sample numbers to a manageable size for more detailed modelling approaches. However, describing shape is complex.

In the fields of Biology and Palaeontology, geometric morphometrics (statistical shape modelling) is an emerging technique [22,24,25]. It can take into account variations in shape (geometric properties) or form (shape and size), help relate performance to form [22], and provide summary metrics and mean shapes. Geometric morphometrics can be applied in two- or three-dimensions [22–26], and can be used alongside finite element analysis to give better insights into mechanical performance [22,23]. Geometric morphometrics could, therefore, be applied to describe changes in shape within a diverse population of rackets, to inform efficient modelling strategies for assessing performance and injury risk. Moreover, how the shape of tennis rackets has changed over time has not been explicitly measured before.

The aim of this study was to describe tennis racket shape in detail using geometric morphometric analysis over a broad range of designs, dating back to the origins of the game. By combining geometric morphometric analysis with principal component analysis, we will summarise how racket shape has changed. We used the dataset of over 500 tennis rackets published by Taraborrelli et al. [10], with a view to capturing more of the variation in racket shape than what they achieved when applying PCA to 12 manual measurements (i.e., > 64% information in ≤ 3 principal components). Using morphometric analyses, we also present mean racket shapes from different material and age groupings, to summarise key racket shape changes. Based on the findings of previous work, we hypothesise that racket shape will change over time, particularly in line with changes in material.

## 2. Methods

Data were collected from 514 rackets from 1874 to 2017 from the Wimbledon Lawn Tennis Museum (n = 412), a brand’s headquarters (n = 90), the International Tennis Federation (n = 4) and Manchester Metropolitan University (n = 8). These were all part of the dataset explored in Taraborrelli et al. [10] and made available in the Supplementary Material. As noted elsewhere [10,27], rackets were selected based on the condition of those available in the collections (e.g. with strings). The earliest year (1874) corresponds to the origins of lawn tennis and hence the oldest rackets available in the museum, and the latest year (2017) corresponds to the newest rackets available at the brands headquarters at the time of data collection. During data collection, rackets were photographed from above on a white sheet to enable silhouettes to be extracted for two-dimensional shape analysis.

Based on the findings of Taraborrelli et al. [10], five year groups, each spanning 29 years, were selected to capture key periods in the history of tennis racket design. It is clear from Taraborrelli et al.’s paper [10] (especially in Figs 5–8) that racket parameters have distinct, discrete groupings over time. Specifically, 1870–1899 was a period of initial experimentation in the design of wooden tennis rackets. From 1900–1959 wood remained as the dominant material, with only small, incremental changes in tennis racket design. 1960–1989 was a period of experimentation with new materials that saw rapid change in tennis racket design, with composites emerging as the dominant material. Since 1990, most tennis rackets have been made from composite materials. Therefore, all the rackets were sorted into five year groupings (1870–1899, 1900–1929, 1930–1959, 1960–1989 and 1990–2019) and three material groupings (wood, other and fibre-polymer composites) for further analysis (Table 1).

**Table 1:** Racket metrics measured.

Metric	Units	Description
*Researched metrics*		
Date	-	The earliest date of the racket release was carried out using Kuebler (11), Wimbledon Lawn Tennis Museum catalogue, as well as manufacturer websites. They were allocated to five groups: 1870–1899, 1900–1929, 1930–1959, 1960–1989 and 1990–2019
Material	-	Visual inspection identified primary racket materials as wood, fibre-polymer composite, or other (including steel, aluminium, and mixtures of metal and wood or metal and composite).
*Measured metrics*		
Racket length	m	Total racket length.
Head length	m	External head length at longest point.
Head width	m	External head width at widest point.
Frame thickness	m	Estimated as half the difference between the external and internal head width.
Frame depth	m	Mean of the minimum and maximum frame depth measurements.
Mass	g	Total mass of the racket using digital scales.
Centre of mass location from the butt	m	Using digital scales to obtain the product of racket length and the ratio of the scale reading to the total mass.
*Moment of inertia (MOI) models detailed in Taraborrelli et al*. (27)		
Transverse MOI	kg m^2^	MOI acting about a lateral in-plane axis passing through the butt.
Polar MOI	kg m^2^	or ‘twistweight’, is the MOI acting about the longitudinal axis of the racket.

**Fig 1:**
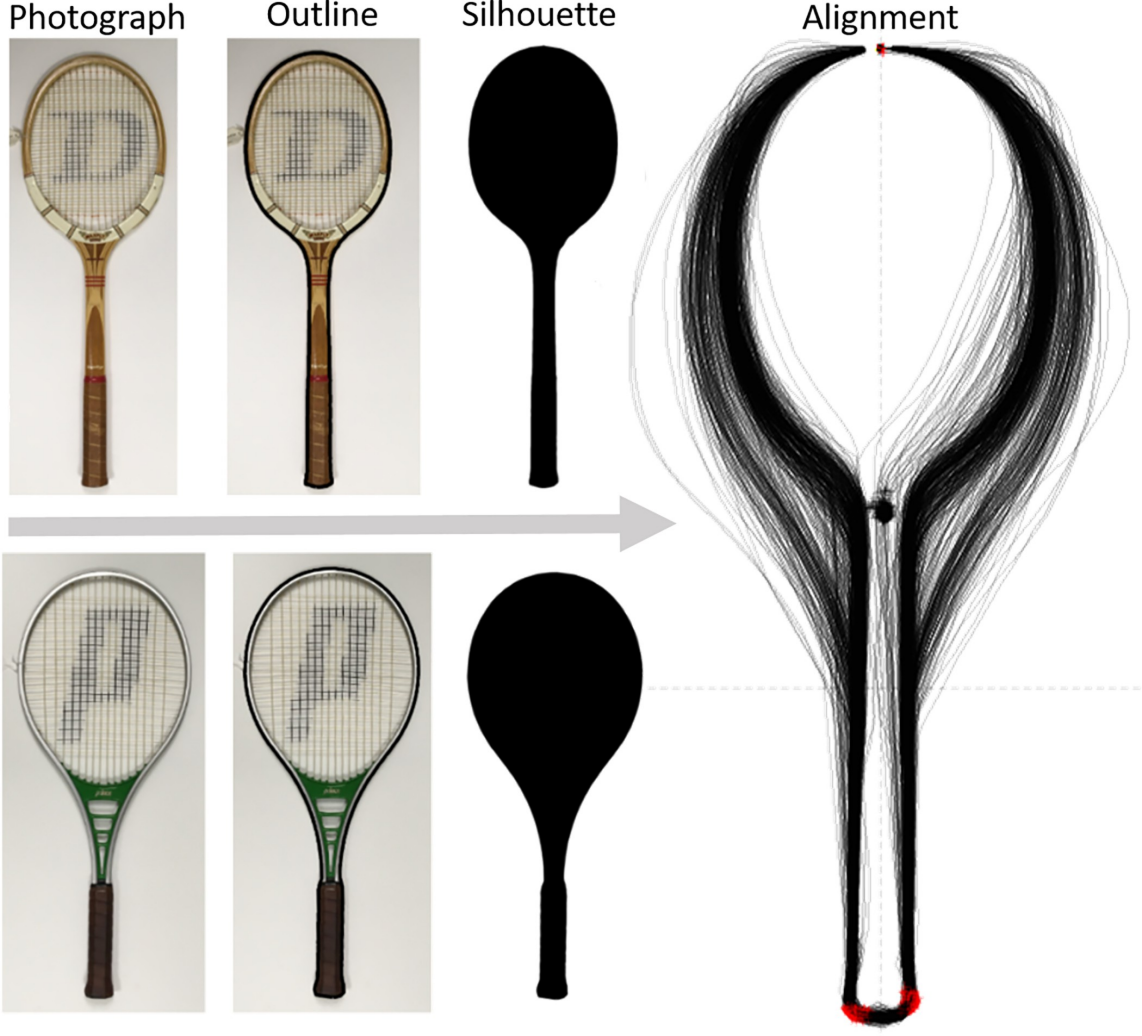
Image processing examples for shape analysis. All rackets were photographed from above. Matlab was used to make an outline and fill for a silhouette image. All silhouette images were then inputted to the R Package Momocs and aligned via a Procrustes alignment on three coordinates (red points). All photographs of the rackets were taken by the authors.

All the photographs were processed using the image processing toolbox in Matlab 2020a, applying the functions *mask*, *imerode* and *imfill*, to extract the outline that was then filled to make a silhouette (Fig 1). These silhouettes did not include internal details, such as whether the racket had an “open” or “closed” throat section. All silhouettes were saved as.jpg files and imported for morphometric analysis using the R Package Momocs [26]. The Momocs package was chosen as it can describe outline shapes without needing many specific landmarks in two-dimensions, such as that of the smooth outline of a racket head from the silhouettes from our photographs.

All racket outlines were extracted (with 1,685 ± 211 coordinates per outline) and aligned using a full generalised Procrustes alignment using three landmark points: left and right base handle points and the top of the head (red points in Fig 1). Outlines were aligned, primarily, by the point at the top of the head, and checked by eye (Fig 1). Outline x and y points were then approximated using elliptical Fourier transforms with seven harmonics. The number of harmonics was automatically calculated to give 99% of the total cumulative harmonic Fourier power, which can be considered as a measure of shape information. These approximated outlines were also checked by eye. Since the harmonic coefficients (4 per harmonic) can be considered as quantitative variables, all were entered into a principal component analysis to give summaries of the racket shapes. As there were seven harmonics, each with four coefficients, there were 28 quantitative variables in total, and hence 28 principal components. Mean racket outlines for the year and material groupings (Table 1) were extracted using the Momocs MSHAPES function on the outline x and y points using elliptical Fourier transforms with 25 harmonics.

The racket metrics were extracted from Taraborrelli et al. [10], where details on the experimental protocol and models can be found. The experiments and models are also summarised in Table 1.

The principal components that captured most of the variation of racket shape (PC1 and Principal Component 2 (PC2)) were investigated in terms of both the year and material groupings (Table 1). Pairwise Pearson’s correlation tests were used to examine associations between the first and second principal components, and the racket metrics (racket length, head length, head width, frame thickness, frame depth, mass and centre of mass location). Bonferroni adjustments were adopted to correct for these multiple comparisons at the p<0.007 significance level. A step-wise regression was also constructed to predict PC1, using the same racket metrics. The standardised residuals from the regression analysis were plotted to confirm that they were distributed normally. Pairwise Pearson’s correlations were also used to explore associations between the principal components and the Transverse and Polar MOIs.

Q-Q plots were examined to confirm that the PC1 variable was normally distributed in each grouping. Between-ANOVAs were then conducted with PC1 as the dependent variable, and the year and material groupings as the independent variables. Significance level was p<0.05 and partial eta squared (η^2^p) was used to quantify the effect sizes, where η^2^p > 0.01 is small, η^2^p > 0.06 is medium and η^2^p > 0.14 is large [28]. 90% confidence intervals were calculated using the NoncF SPSS Sytax Code of Wuensch [29].

## 3. Results

**Fig 2:**
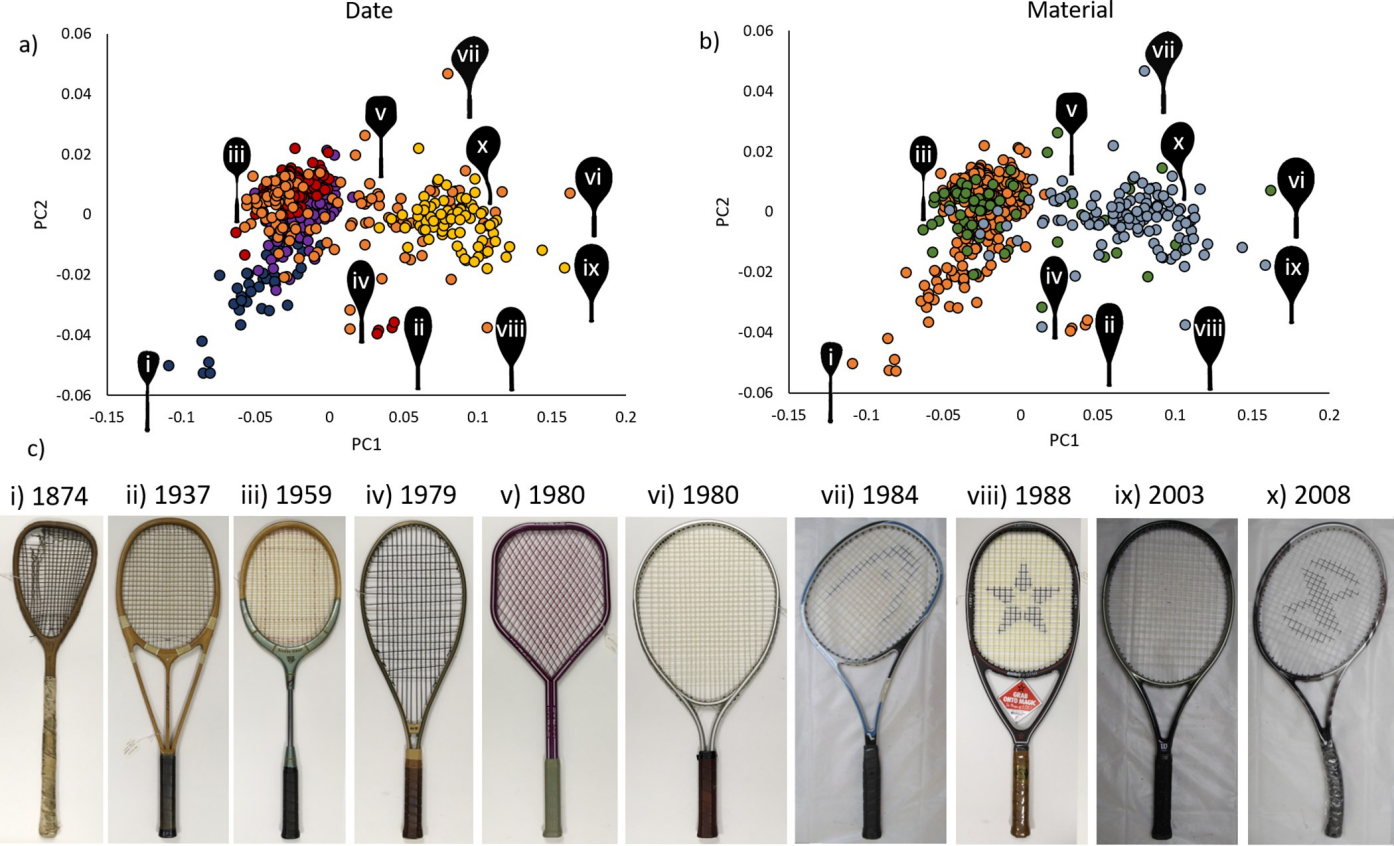
Summary of racket morphometric principal component measures PC1 and PC2. Panels a) and b) show scatterplots of PC1 and PC2. Panel c) shows examples of rackets towards the edge of the PC1 and PC2 distribution, with unusual shapes: i) Unknown brand, Sphairistike, 1874; ii) Hazells, Streamline White Star, 1937; iii) Grays of Cambridge, Silver Gray, 1959; iv) Kuebler & Co., Plus 60, 1979; v) Inter Business AG, Myrac, 1980; vi) Weed USA, Weed, 1980; vii) Snauwaert, Ergonom Graphite, 1984; viii) Chris New Tech Sports Ltd, CTE 5 Star Power G, 1988; ix) Wilson, Triad 2.0 Hammer, 2003; x) Neoxx, ST 285, 2008. Scatterplot markers are coloured with date (left panels) and material (right panels) groupings. For date, blue is 1870–1899, purple is 1900–1929, red is 1930–1959, orange is 1960–1989 and yellow is 1990–2019. For material, orange is wood, green is other and blue/grey is composite. All photographs of the rackets were taken by the authors.

Together PC1 (86.9%) and PC2 (5.4%), from the 28 principal components, captured over 90% of the variation in racket shape, and these are presented in Fig 2.

### Racket shape and metric associations

PC1 was correlated to racket head width, head length, mass, frame depth and length (Table 2, with decreasing r-values). Since head width and length were both strongly correlated to PC1 (r>0.80, Table 1), head size is likely to be associated with PC1. This is further supported by the example racket silhouette shapes in Fig 2A and 2B that show head size tended to increase with values of PC1. When all the racket metrics were added to a stepwise linear regression model, racket head width and length, mass, frame depth and racket length all significantly improved the model, and strongly predicted PC1 (r^2^ = 0.878, p<0.001, Table 2).

**Table 2:** Statistical tests of association with PC1, including a Pearson’s correlation and step-wise linear regression.

PC1 (n = 514)	Head width (m)	Head length (m)	Mass (g)	Frame depth (m)		Racket length (m)	Frame thickness (m)	Centre of mass (m)
**Pearson’s Correlation**								
**r**	0.916**	0.801**	-0.528**	0.410**		0.187**	-0.064	-0.038
**p**	<0.001	<0.001	<0.001	<0.001		<0.001	0.149	0.387
**Stepwise Regression: r**^**2**^ **= 0.878, p<0.001**								
**t**	25.408**	11.270**	-2.563*	4.030**		-6.537**	-	-
**p**	<0.001	<0.001	0.011	<0.001		<0.001		

** in the Pearson’s correlation row corresponds to p<0.007, which is significant with a Bonferroni adjustment for multiple comparisons. ** in the Stepwise Regression row corresponds to p<0.001

* corresponds to p<0.05, and—corresponds to that variable not being included in the regression as it did not significantly improve the model.

PC1 was also significantly correlated to Polar (Pearson’s correlation: r = 0.862, p<0.001) and Transverse (Pearson’s correlation: r = -0.506, p<0.001) MOI.

PC2 only captured 5.4% of the variation in the data, and it was not correlated to any of the racket metrics (i.e. all r values < 0.3 and all p values > 0.05), therefore it will only be qualitatively discussed from hereon in.

### Racket shape changes with date and material

PC1 showed clear transitions in racket shape, progressing from left to right across the x-axis in Fig 2A and 2B. Overall, lower values of PC1 occurred in older, wooden rackets with smaller heads, while higher values occurred in newer, composite rackets with larger heads (Fig 2A and 2B). Indeed, PC1 was significantly affected by both racket age (date: F(4,513) = 10.055, p<0.001, η^2^p = 0.074, 90% Confidence Interval: 0.0356–0.1052) and material (F(2,513) = 4.236, p = 0.015, η^2^p = 0.017, 90% Confidence Interval: 0.0018–0.0356). Head width and length tended to increase with PC1; however, the shape of the rackets was diverse and not just a simple association with head size, as illustrated by the example silhouettes in Fig 2A and 2B. Associations of PC2 were less clear (Fig 2A and 2B), although more unusually-shaped rackets, i.e. those that were asymmetric (Fig 2C i, vii, x), pentagonal (Fig 2C v) or with extended string beds (Fig 2c iv), tended to have more extreme values of PC2 (Fig 2A and 2B).

**Fig 3:**
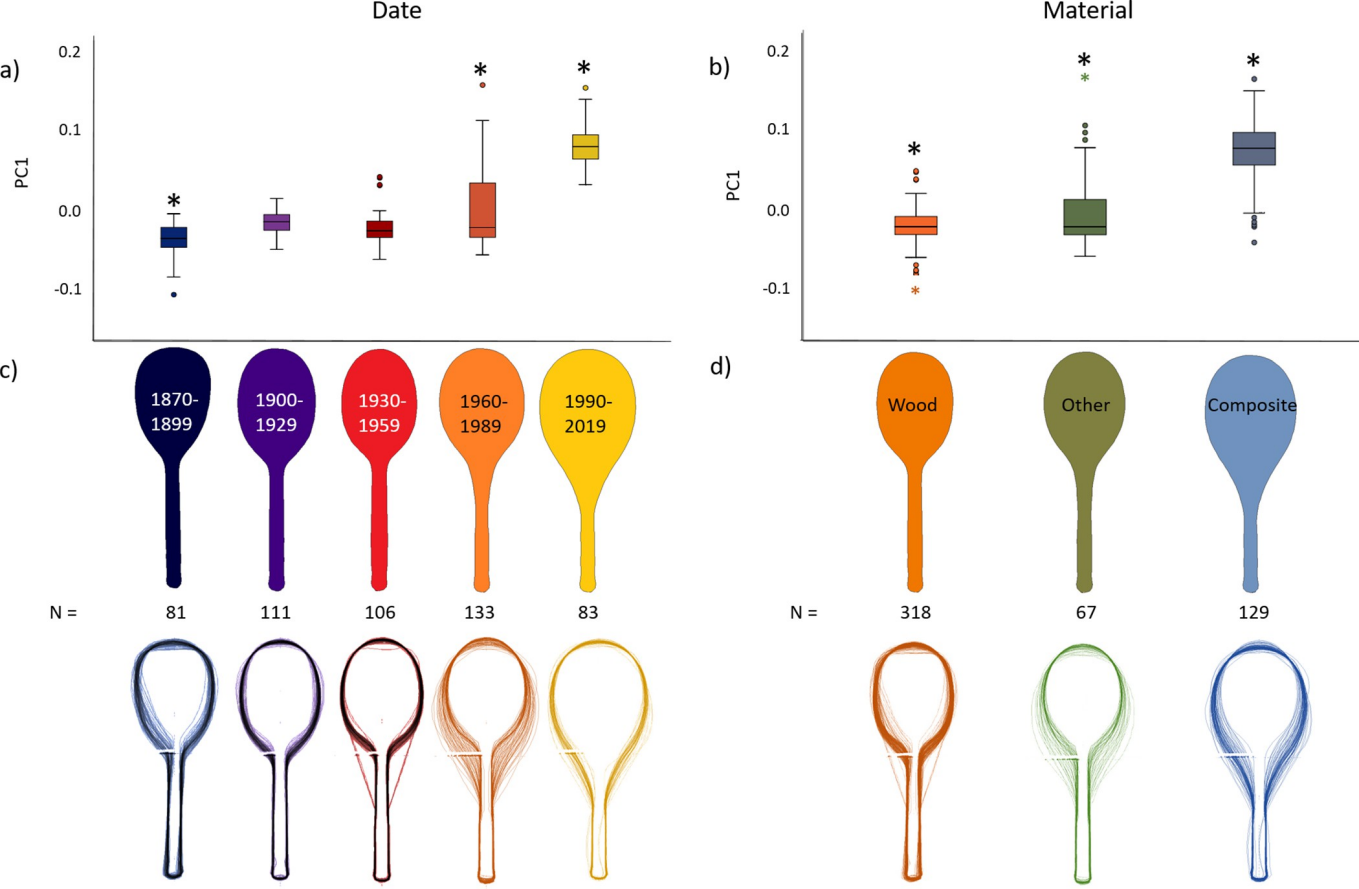
Summary of racket shape changing with date and material. Panels a) and b) show boxplots of the principal component measure, PC1, with significant differences (p<0.05) indicated by asterisks (*). Panel c) shows the mean shape of the rackets in each grouping (top) and a stack of aligned silhouette outlines for all rackets in that grouping (bottom).

Racket age had a medium effect on PC1 (η^2^p = 0.074), with the shape of those of 1870–1899 being significantly different to those of 1960–1989, which were significantly different to those of 1990–2019 (Fig 3A). In the scatterplot of PC2 vs. PC1 (Fig 2A), 1870–1899 rackets occupied the bottom left-hand corner with both low PC1 and PC2 values. In contrast, 1990–2019 rackets occupied the right hand side of the plot with high PC1 and intermediate PC2 values. 1900–1929 and 1930–1959 rackets occupied a similar area on the left hand side with low to intermediate PC1 and PC2 values. 1960–1989 rackets were the most varied in terms of their shape with PC1 and PC2 spanning low and high values. These patterns are further supported by the mean racket shapes constructed during the morphometric analysis, and the aligned silhouette outlines (Fig 3C). 1870–1899 rackets had small heads and long handles, and were often asymmetric. 1900–1929 and 1930–1959 rackets were of similar shape, with small heads and long handles. Rackets from 1960–1989 had larger heads, which were also more oval due to a more “open” throat region, and had many, varied shapes (see orange silhouette outlines in Fig 3C). Rackets from 1990–2019 had even larger and more oval-shaped heads. Indeed, the racket heads appear to get more “egg-shaped” from 1870–2019, with the string bed and throat region extending further down the handle (Fig 3C).

Material had a small (η^2^p = 0.017) effect on PC1. Wooden rackets had significantly lower PC1 values than the other material (mainly metal) rackets, which had significantly lower PC1 values than the composite rackets (Fig 3B). In the scatterplot of PC2 vs. PC1 (Fig 2B), wooden rackets occupied the left hand side of the plot, spanning low to intermediate values of both PC1 and PC2. Other material rackets mainly occupied a similar space to the wooden rackets, with intermediate PC2 and low to high PC1 values. Composite rackets occupied the right hand side of the plot and tended to have high PC1 and intermediate PC2 values. These patterns are also further supported by the mean racket shapes for each material, and the aligned silhouette outlines (Fig 3D). Wooden and other material rackets had similar shapes, with small heads and long handles. In contrast, composite rackets had larger heads, which were also more oval, or egg-shaped.

## 4. Discussion

We demonstrate here that geometric shape analyses and PCA can capture over 90% of the variation in racket shape (PC1: 87%, PC2: 5%), which is much higher than the amount obtained previously using discrete, manual racket measurements (64% with 3 principal components [10]). It is also possible to capture key racket summary shapes using geometric analyses to generate mean shapes (Fig 3). In agreement with our original hypothesis, our findings suggest that historical developments in the materials used to make tennis rackets have had a significant, but small, effect on their shape (Fig 3B). While this finding supports our hypothesis, the age of a racket had a larger effect (medium effect size) on its shape than the material it was made from. So, while new materials may have been a catalyst that led to changes in racket shape, developments were somewhat gradual and incremental, occurring slowly over time; these could have been influenced by many factors, like player preference, consumer trends, and differences between brands [10]. Indeed, racket age and material are not independent from each other, with the materials, and associated tools and manufacturing processes, available to the engineer changing over time. Therefore, we observe gradual changes in tennis racket design overall, which are typical of the, often incremental, nature of the product design process.

### Tennis racket shape has changed over time

The first tennis rackets had small heads and were often asymmetric. In the 1900–1959 groups, rackets were usually symmetric, but still had small heads. The period of experimentation from 1960–1989 involved more diverse shapes (note the spread of the orange data points in Fig 2A and the variety of orange outline shapes in Fig 3C), with an overall tendency towards rackets with larger and more ovoid, or egg-shaped, heads. Racket heads in the period 1990–2019 were larger and more ovoid than in the older rackets, as expected [6,9–12]. Both Taraborrelli et al. [10] and Miller [12] have proposed that increases in racket head size were driven by innovations in material. Miller [12] states that the stress limitations of wood limited the size of the racket head, and that the development of new, stronger composite materials allowed for larger head sizes. Composite rackets are also lighter [10], which accounts for the significant negative correlation observed between PC1 and mass (Table 2).

While previous studies have noted the overall increase in racket head size [6,10], morphometric analyses can go further by analysing the whole shape of the racket. Taraborrelli et al. [10] were only able to account for about two thirds of the information in the dataset, with the first three principal components, when they applied PCA to 12 measurements of each racket, whereas we captured almost 90% of the variation with PC1 alone, when pairing morphometric analysis with PCA. As well as capturing a good degree of racket shape variation, analysing images is also more efficient than taking manual measurements. Our mean racket shapes, and the aligned silhouette outlines, show that racket head size has not only got larger, but also more ovoid, or egg-shaped. This means that the widest part of the racket can be further from the handle than if the head was circular. Moreover, it means that the head and throat region also extend further down the handle.

### Implications for play

The larger and more oval heads of modern rackets are likely to affect the racket’s MOI [30], and hence racket performance and “feel”. Polar and Transverse MOI were both correlated to racket shape (PC1) (r = 0.862 and 0.506, respectively). Polar MOI is associated with “twist weight” and quantifies resistance to rotation for impacts away from the longitudinal axis of the racket [30]. As first noted by Brody [30], Taraborrelli et al. [27] showed that racket width was well-correlated to Polar MOI (r = 0.893), and newer, composite rackets (Fig 3 in [27]) tend to be wider. Wider composite rackets are, therefore, more resistant to rotation about the longitudinal axis and are more ‘stable’ during play.

Transverse MOI can influence ball speed off the racket [31]. It is also associated with the ‘swing-weight’, which captures how hard it is to accelerate the racket through a swing [32–34]. Taraborrelli et al. [27] showed racket mass to be the largest predictor of Transverse MOI (partial correlation = 0.970), followed by the centre of mass location (partial correlation = 0.906). This finding indicates that the rackets were more varied in terms of their mass than their centre of mass location (coefficient of variation of 10% for mass vs. 5% for centre of mass location). Since composite rackets are lighter [6,10], they have lower Transverse MOI and hence lower swing-weight. The association of swing-weight with racket performance is complex, and previous research has found that rackets with lower swing-weights can be swung faster when serving [35,36]. It was this faster swing speed that led Haake et al. [6] to conclude that a player could serve faster with a lightweight, modern racket. A combination of racket shape and mass, driven by composite material development, has likely enabled the increase in performance and speed of play that we see in tennis today.

**Fig 4:**
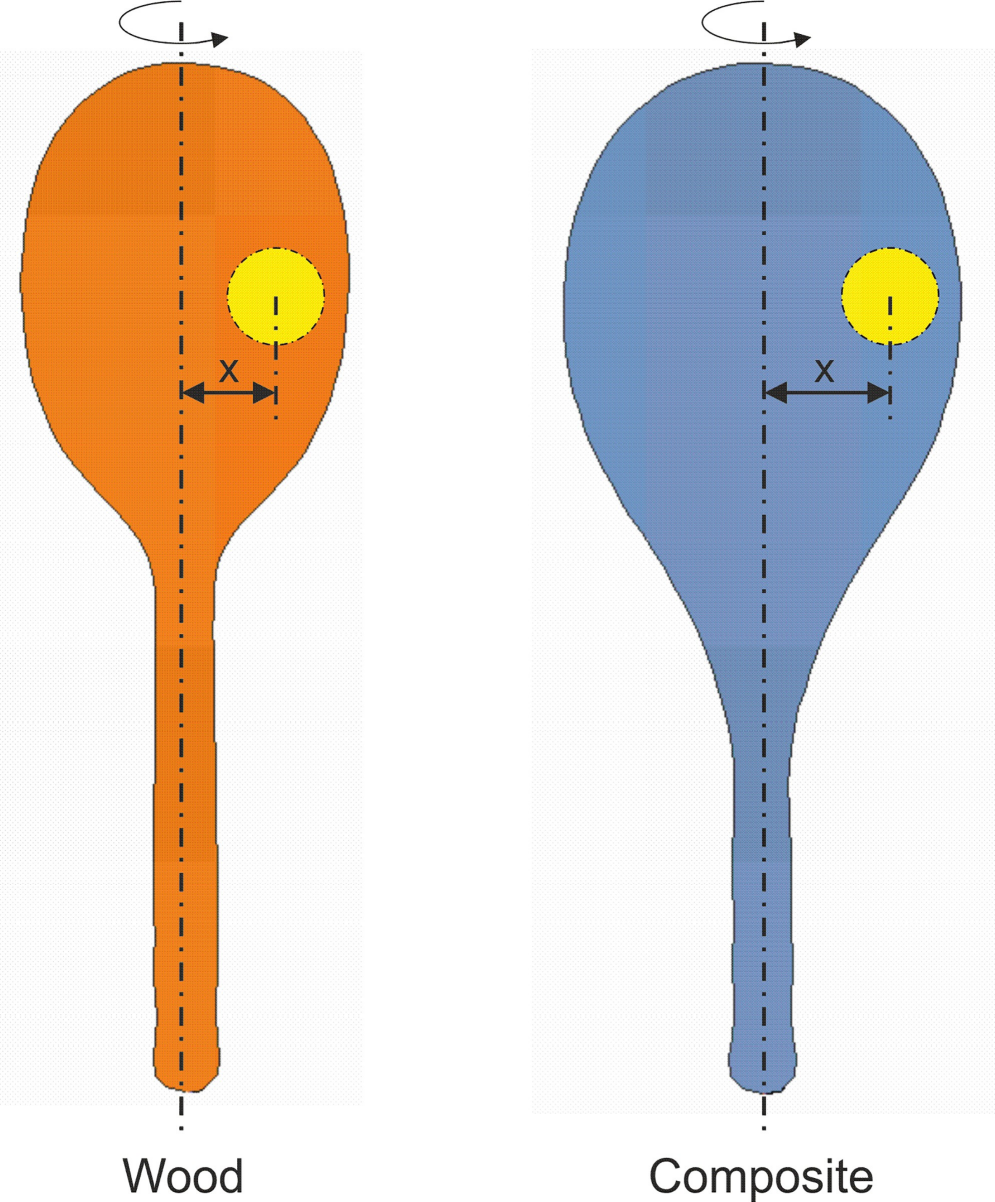
Illustration using mean racket shapes from morphometric analysis to demonstrate how the ball can be hit further off-centre (distance x) in a composite racket.

An increase in speed of play is also associated with less accurate shots, with the ball often landing further from the centre of the string bed when the racket is swung faster [35]. It is also worth bearing in mind that the larger head sizes mean that, especially for beginners, the ball can impact further from the longitudinal axis in wider newer rackets than in narrower older rackets (see Fig 4). Not only this, but since the newer rackets are egg-shaped, the widest point of the racket can be further from the grip than if the racket head was circular, or elliptical. A ball impacting off-centre at the widest point of the string bed will cause both lateral and longitudinal rotation of the racket, forcing the wrist to extend [16]. Off-centre impacts that rotate the racket about its longitudinal axis can also cause radial movement at the wrist and reduce shot accuracy by changing the rebound angle of the ball [37]. Composite rackets are lighter, with a lower Transverse MOI, and their centre of mass location can vary between designs [6]. It is unknown whether the higher Polar MOI from the wider head can compensate for misplaced hits that can be further from the centre of the string bed, particularly when both the incoming ball and lighter racket are moving faster. As suggested by Miller [12], the shape of newer rackets might have implications for injury, which warrants further investigation. The mean racket shapes for the material and age groupings, and the aligned silhouette outlines (Fig 3C and 3D), could support the development of finite element models of typical, and less common, designs from different eras to facilitate such an investigation. Players suffering from upper extremity injuries may even consider using narrower and heavier rackets, so they can make slower shots that are closer to the centre of the string bed. Alternatively, they could add a few grams of lead tape at the widest points of the head, to increase the mass and Polar MOI of their racket, and move the centre of mass closer to the centre of the string bed [27,38].

### Further racket shape complexities

Here, we have mainly described the overall, or mean, patterns of the rackets shapes that we have quantified. However, racket shapes are diverse, especially in the period of design and material experimentation from 1960 to 1989 (see orange outlines in Fig 3C). For instance, asymmetric rackets were common in the 1870s, but also made appearances more recently, including in 1984 and 2008 (see example shapes in Fig 2C i, vii and x). Indeed, the Neoxx ST 285 asymmetric racket from 2008 (Fig 2C x) was marketed to be more ergonomic. While an asymmetric design does not seem to persist in our samples for long, the recurrence of this theme makes it an interesting shape to investigate further. Other innovative racket designs can also be seen in Fig 2C, and include a string bed extending to the handle region (Fig 2C iv, v) and unusual head and throat shapes (Fig 2C ii, iii, v, viii). The effect of these innovative shapes on racket performance cannot be captured using beam models. We recommend that shape analyses, such as morphometrics, and engineering simulation techniques, such as finite element analysis, should be applied to investigate the effect of racket shape on mechanical properties. Researchers could use the mean shapes in Fig 3 as a basis for initial inputs when modelling rackets from different eras, with the aligned silhouette outlines used to inform further investigations into less common designs. Such analyses could cover both the frequency and shape of vibration modes [20] and the impact with the ball [17–19], with a view to improving our understanding of the effect of racket shape on both performance and injury risk. As well as modelling, commercial sensors are now available for analysing tennis strokes [10,39]. These sensors have the capacity to report parameters like speed and impact location. Trainers and clinicians could use these sensors to monitor injury risks and causes in a variety of rackets, which would also provide field data to inform modelling strategies.

### Limitations

This work demonstrates how morphometric analysis can be applied to summarise trends in shape within a large and diverse sample of sports equipment; in this example, 514 tennis rackets dating from 1874 to 2017. However, this work is not without limitations. The rackets included here do not account for every design that has ever been made, and the number of samples varied by year and brand. Indeed, the rackets included here were limited to those available in the collections at the time of data collection. The newest samples from 2017 are now a few years old and do not necessarily represent the latest designs. There was also uncertainty in determining the age of some of the rackets, particularly the oldest and rarest samples, with estimates expected to be accurate to within five years [10]. The biggest limitation, however, was that the shape analysis was only in two-dimensions, determined from a silhouette extracted from a photograph of the racket taken with the image plane parallel to the string bed. As such, the analysis did not capture the depth of the racket, which can vary between designs and is a key factor in determining bending stiffness [8]. The frame thickness around the head and throat region could also not be examined here, as the Momocs analysis technique only uses silhouette images.

### Future work and wider implications

Future work could, therefore, explore the application of three-dimensional geometric morphometrics to sporting equipment, and even look to combine this with finite element analysis [22,23]. Such an approach would not be straightforward since it requires the three-dimensional geometry of each racket to be used as an input for the analysis. While it may be possible to obtain computer aided design geometry for modern rackets from the developers, another technique would be required to obtain the geometry of older samples, such as by laser scanning, or recreating the geometry in a computer aided design package from manual measurements. An alternative, lower cost and less time-consuming option could be to create a simplified computer aided design geometry of the racket, by taking the two-dimensional geometry extracted from a photograph and extruding it by the measured thickness. The feasibility of such an approach for efficiently recreating the geometry of tennis rackets for three-dimensional morphometric analysis could be explored in further work.

The work presented could have various practical applications beyond fundamental research, including for tennis players, coaches, equipment brands, regulatory bodies (International Tennis Federation (ITF)), museums/collections and educators. The ability of geometric morphometric analysis to objectively identify shape groupings, outliers and mean shapes will streamline future modelling approaches, benefitting both equipment brands and the ITF. Specifically, they will be able to focus their efforts on one representative shape of a group of rackets, rather than producing many, geometrically faithful models. Through observations of the racket shapes in Figs 2 and 3, players and coaches could gauge how the shape of a particular design compares to others of a similar age or material. In a similar manner, these figures may also help: i) equipment brands to identify opportunities for new and innovative racket shapes; ii) the ITF in devising strategies for furthering our understanding of how racket design influences the game; and iii) museums and collection to identify when they have located a particularly rare or unusually shaped racket. Indeed, we feel that an area where the work could be particularly beneficial is engineering education [40], with the history of tennis racket design offering an interesting case study for educators [8] when teaching topics like mechanics and finite element modelling, and when looking to enthuse and inform people about science and engineering during outreach and public engagement activities. Furthermore, the diversity of tennis racket shapes and the availability of racket photographic datasets (such as from equipment brand websites, in books [9,11] and museum catalogues) makes tennis rackets an excellent case study for further developing geometric morphometric analyses and other shape modelling approaches.

## 5. Conclusion

We suggest that shape is an important parameter in modelling equipment performance and mechanics, and should not be ignored in favour of simple beam models. We propose that investigating the effect of racket shape on performance might lead to some interesting innovations away from the popular trend of increased head size and decreased mass in symmetrically-shaped rackets. Using finite element analysis as a research and design tool in conjunction with morphometrics will be a succinct way to develop and test new sports equipment designs, and should form a focus of future research. While it is inefficient to develop a finite element model of every possible racket, we demonstrate here that morphometrics can be used to capture mean rackets shapes, outliers and key shape changes that can then be targeted further with in-depth finite element analysis. Combining geometric morphometrics with finite element analysis will provide a powerful tool for developing our understanding of equipment mechanics and design, as well as its effect on biomechanics. The application of such engineering techniques could allow us to improve on the modern racket shape that has persisted since the 1990s.
